# Curcumin, A Polyphenolic Curcuminoid With Its Protective Effects and Molecular Mechanisms in Diabetes and Diabetic Cardiomyopathy

**DOI:** 10.3389/fphar.2018.00472

**Published:** 2018-05-09

**Authors:** Jia Zheng, Jing Cheng, Sheng Zheng, Qianyun Feng, Xinhua Xiao

**Affiliations:** ^1^Department of Endocrinology, Key Laboratory of Endocrinology, Ministry of Health, Peking Union Medical College Hospital, Diabetes Research Center of Chinese Academy of Medical Sciences and Peking Union Medical College, Beijing, China; ^2^The Key Laboratory of Cardiovascular Remodeling and Function Research, Chinese Ministry of Education and Chinese Ministry of Health, The State and Shandong Province Joint Key Laboratory of Translational Cardiovascular Medicine, Shandong University Qilu Hospital, Jinan, China; ^3^Graduate School, Tianjin University of Traditional Chinese Medicine, Tianjin, China

**Keywords:** curcumin, curcuminoids, diabetes mellitus, diabetic cardiomyopathy, inflammation, antioxidant, apoptosis

## Abstract

As the leading cause of morbidity and mortality in patients with diabetes, diabetic cardiomyopathy (DCM) imposes enormous burden on individuals and public health. Therapeutic regimes for DCM treatment have proven to be challenging, with limited efficacy, low compliance, and potential adverse effects. Curcumin, as the most active compound derived from the root of turmeric, exhibits strong anti-inflammation, antioxidant, and anti-apoptosis properties. Recently, clinical trials and preclinical studies have shown that curcumin exerts protective effects against a variety of diseases, including diabetes and its cardiovascular complications. In this review, the clinical trials about curcumin supplementation on diabetes and DCM are presented, and the specific mechanisms by which curcumin might mitigate diabetes and DCM are fully discussed. A better understanding of the pharmacological role of curcumin on diabetes and DCM can provide clinical implications for the intervention of the onset and development of diabetes and DCM.

## Introduction

The prevalence of diabetes is increasing rapidly during the last three decades, which is becoming one of the most epidemic non-communicable diseases. The International Diabetes Federation [IDF] (2017) estimates that the number of people with diabetes reaches 425 million worldwide in 2017 and it will rise to 629 million by 2045, indicating a 45% increase throughout the world. Globally, about one in eleven adults have diabetes, and 90% of whom are diagnosed as type 2 diabetes mellitus (T2DM). T2DM and its complications have contributed tremendously to the burden of mortality and health cost worldwide. Among the various complications of diabetes, cardiovascular complications are believed to be the leading causes of disability and death among diabetic patients, particularly for diabetic cardiomyopathy (DCM) (Cai and Kang, 2003). Cardiovascular diseases (CVDs) typically develop about 15 years earlier in T2DM (Booth et al., 2006), and T2DM patients are more than twice as likely to develop CVDs as compared with those without T2DM (Sarwar et al., 2010).

## Limitations in Therapeutic Strategies for Diabetes and Dcm

In 2016, the Global Burden of Diseases (GBD) study reported that T2DM and its complications accounted for a 22% increase in disability-adjusted life years (DALYs) during the last decade (2016), imposing enormous burden on individuals and public health (GBD 2015 Risk Factors Collaborators, 2016). Despite prominent advances in diabetes prevention, treatment, glucose monitoring and novel glycemic control biomarkers, detrimental cardiovascular complications, especially for DCM still remain rigorous in patients with T2DM (Pirola et al., 2010). Nowadays, therapeutic regimes for diabetes and DCM include several clinical managements, involving lifestyle modifications (diet and exercise), glucose and lipid control (antidiabetic and lipid-lowering drugs), hypertension treatment, and coronary artery diseases intervention. The commonly used therapeutic strategies for CVDs in diabetic patients include cardiac glycoside, Ca^2+^ antagonist, β-adrenergic blocking agents, angiotensin converting enzyme inhibitor (ACEI), angiotensin receptor blocker (ARB) and diuretics (Marwick et al., 2018). However, the incidence and mortality rate of DCM still remains high, it is imperative to develop novel and effective therapeutic strategies for diabetes and DCM.

## Curcumin and Its Protective Effects on Human Health

**FIGURE 1:**
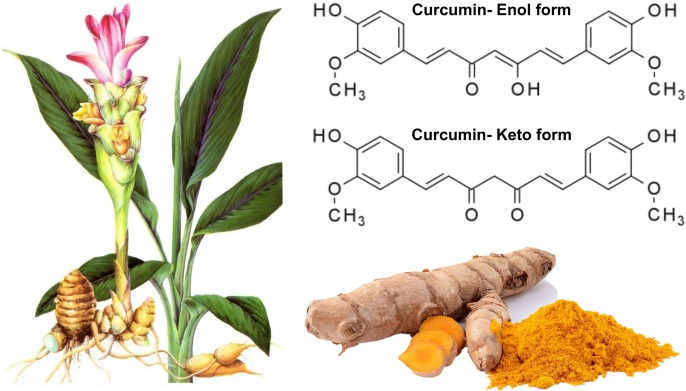
The molecular structure of curcumin isolated from the root of turmeric. Curcumin, a natural compound, is the most active agent of the polyphenolic curcuminoids derived from the root of turmeric (Curcuma longa). It is a tautomeric compound existing in organic solvents as its enolic form, and in water as a keto form. Turmeric, as a member of the ginger family, has been widely used as an herbal medicine, ingredient of cosmetics, and dietary supplement.

Plants and herbs have historically been widely used for medicinal purposes. Traditional Chinese Medicine (TCM), with a history of more than 2,000 years, includes various forms of herbal medicine and dietary therapy (Hao et al., 2015b). Natural plants and their active derivatives have been deemed as novel therapeutic agents for multiple diseases, such as CVDs (Hao et al., 2017), metabolic disorders (Martel et al., 2017), rheumatic autoimmune diseases (Dahan et al., 2017), and cancer (Seca and Pinto, 2018). A randomized, placebo-controlled study indicated that dietary supplementation with equol and resveratrol can reduce the severity of menopausal symptoms in recently postmenopausal women, that can improve menopause-related quality of life in healthy women (Davinelli et al., 2017). Curcumin, a natural compound, is the most active agent of the polyphenolic curcuminoids derived from the root of turmeric (Curcuma longa). It is a tautomeric compound existing in organic solvents as its enolic form, and in water as a keto form (Manolova et al., 2014) (**Figure 1**). Traditionally, turmeric, as a member of the ginger family, has been widely used as an herbal medicine, ingredient of cosmetics, and dietary supplement (food flavoring and coloring). In addition to a dietary ingredient, turmeric is also prescribed abundantly for ailments in traditional medicine (Nelson et al., 2017). Numerous studies suggest that curcumin is a potent molecule that can exert a variety of positive pharmacological effects, including anti-inflammation (Sikora et al., 2010; Koeberle and Werz, 2014), antioxidant (Nakmareong et al., 2011), and anti-apoptosis (Topcu-Tarladacalisir et al., 2013) properties. Corbi et al. (2016) also showed that curcumin can exert relevant immunomodulatory and/or anti-inflammatory activities in the context of brain aging. Some phytochemicals, such as curcumin inducing increase in nuclear factor (erythroid-derived 2)-like 2 (Nrf2) and sirtuin 1 (SIRT1) activity could be able to inhibit the nuclear factor kappa-B (NF-κB) activation and then to end the progression of the brain aging (Corbi et al., 2016). In recent years, compelling data indicates that curcumin is a protective compound against insulin resistance (Yekollu et al., 2011), obesity (Hariri and Haghighatdoost, 2018), diabetes mellitus (Arivazhagan et al., 2015), and CVDs (Hashemzaei et al., 2017; Jiang et al., 2017). However, although the evidence alludes to protective effects of curcumin on human health, information about the effect of curcumin on diabetes and DCM is limited. It is speculated that curcumin may be a pleiotropic molecule targeting diabetes and DCM, with a rather diverse array of metabolic, cellular, and molecular activities. Therefore, the current review aimed to provide an overview of the effect of curcumin on diabetes and DCM, and the molecular mechanisms of curcumin in alleviating diabetes and DCM.

## Molecular Mechanisms Underlying the Pathogenesis of Diabetes and Dcm

Diabetic cardiomyopathy, as a severe complication of diabetes, is characterized by cardiac structure and function disorders (Bugger and Abel, 2014), including metabolic deregulation, left ventricular dysfunction, and myocardial cell deterioration (Marwick et al., 2018). DCM is associated with impaired systolic and diastolic functions with prolonged contraction and relaxation duration, and depressed myocardial contractility and relaxation (Boudina and Abel, 2007). Echocardiography revealed shorter left ventricular ejection duration, and increased wall stiffness in diabetic patients (Poirier et al., 2001). The pathogenesis of diabetes and DCM is multifactorial, and evidence indicates that the risks of diabetes and DCM are not limited to traditional factors, such as atherosclerosis, hypertension, and coronary diseases (Boudina and Abel, 2007). To date, a range of molecular and cellular mechanisms have been proposed for the development and progress of diabetes and DCM, including advanced glycation end products (AGEs) accumulation (Goldin et al., 2006), inflammation activation (Diamant et al., 2005), increased oxidative stress (Anderson et al., 2009), higher induction of apoptosis (Frustaci et al., 2000), and impaired autophagy (Nakai et al., 2007).

## Clinical Trials With Curcumin in Diabetes and Its-Related Cardiovascular Risks

Accumulating clinical trials have revealed that curcumin has its beneficial effects on rheumatoid arthritis (Mande et al., 2016), inflammatory bowel disease (Taylor and Leonard, 2011), cancer (Shehzad et al., 2013; Naksuriya et al., 2014), Alzheimer’s disease (Hathout et al., 2017), and other diseases (Gupta et al., 2012). However, few studies have been conducted to investigate the effects of curcumin on diabetes and its-related CVDs. Importantly, pharmacological research reveal that curcumin is effective, safe, and without toxicity (Prasad et al., 2014). Dyslipidemia is an established factor and increase the susceptibility of atherosclerotic heart disease in diabetic patients. Panahi et al. (2017b) aimed to examine an anti-atherosclerosis effect of curcumin in diabetic patients. It showed that curcuminoids supplementation (1,000 mg/day) for 12 weeks can reduce serum levels of atherogenic lipid levels, including non-high density lipoprotein (HDL) and lipoprotein(a) [Lp(a)] in patients with T2DM (Panahi et al., 2017b). Chuengsamarn et al. (2014) reported that curcuminoids intake (1,500 mg/day) for 6 months continuously increased insulin sensitivity, decreased pulse wave velocity, triglyceride level, and atherosclerosis incidence in patients with T2DM, indicating that curcuminoids supplementation could contribute to a lower risk of cardiovascular events in dyslipidemic patients with T2DM (Chuengsamarn et al., 2014). A report from the same group found that curcumin intervention (1,500 mg/day) for 12 months significantly decreased the incidence of T2DM in pre-diabetic individuals, with a lower level of insulin resistance and higher adiponectin level (Chuengsamarn et al., 2012). A recent clinical trial showed that curcumin supplementation (1,000 mg/day) for 12 weeks exhibited higher adiponectin level and lower leptin concentration, with decreased leptin/adiponectin ratio (a measure of atherosclerosis) in patients with T2DM (Sahebkar et al., 2018). In addition, Panahi et al. (2017a) showed that curcuminoids supplementation (1,000 mg/day) for 8 weeks significantly increased serum total antioxidant capacity (TAC) and superoxide dismutase (SOD) activities, with reduced malondialdehyde (MDA) concentrations in diabetic patients (Panahi et al., 2017a). Since dyslipidemia, insulin resistance, and oxidative stress principally increases the risks of CVDs in diabetic patients, these studies suggest the protective role of curcumin on diabetes and its-related cardiovascular risks. However, clinical trials about the direct effects of curcumin on DCM in patients with diabetes are needed. The clinical studies about curcumin and its protective effects against diabetes and its-related cardiovascular risks are listed in **Table 1**.

**Table 1:** Clinical trials about curcumin and its protective effects against diabetes and its-related cardiovascular risks.

Subjects included	Treatments	Metabolic effects	Reference
118 subjects with T2DM	Curcuminoids (1,000 mg/day) for 12 weeks	- Reductions in serum total cholesterol, non-HDL-C and Lp(a) levels	Panahi et al., 2017
		- Elevations in serum HDL-C levels	
240 patients with T2DM	Curcuminoids (1,500 mg/day) for 6 months	- Reduced pulse wave velocity	Chuengsamarn et al., 2014
		- Increased level of serum adiponectin and decreased level of leptin	
		- Reduced levels of HOMA-IR, triglyceride, uric acid, visceral fat, and total body fat	
240 pre-diabetic individuals	Curcuminoids (1,500 mg/day) for 12 months	- Decreased the number of pre-diabetic individuals who eventually developed T2DM	Chuengsamarn et al., 2012
		- Better overall function of β-cells, with higher HOMA-β and lower C-peptide	
		- A lower level of HOMA-IR and higher adiponectin	
118 patients with T2DM	Curcuminoids (1,000 mg/day) for 12 weeks	- Higher adiponectin level	Sahebkar et al., 2018
		- Lower leptin concentration	
		- Decreased leptin/adiponectin ratio	
		- Elevated serum ghrelin level	
118 subjects with T2DM	Curcuminoids (1,000 mg/day) for 8 weeks	- Elevation in serum TAC and SOD activities	Panahi et al., 2017
		- Reduced MDA concentration	

*DCM, diabetic cardiomyopathy; T2DM, type 2 diabetes mellitus; Lp(a), lipoprotein(a); HDL-C, high density lipoprotein cholesterol; HOMA-IR, homeostasis model assessment-insulin resistance; HOMA-β, homeostasis model assessment-β; TAC, total antioxidant capacity; SOD, superoxide dismutase; MDA, malondialdehyde.*

## Preclinical Studies About Curcumin and Its Effects on Diabetes and Dcm

### Curcumin and Its Anti-inflammatory Effects on Diabetes and DCM

Inflammation plays a critical role of the development of diabetes and its complications (Pereira and Alvarez-Leite, 2014), and inflammation with increased cytokine levels is shown to be closely related to the onset and development of diabetes and DCM (Xu et al., 2003). It demonstrated that intra-myocardial inflammation in DCM is associated with increased macrophages and leucocytes infiltration, significant increment of vascular cell adhesion molecule-1 (VCAM-1) and intercellular adhesion molecule-1 (ICAM-1) expressions, as well as elevated expression of inflammatory cytokines, including interleukin-6 (IL-6), tumor necrosis factor-α (TNF-α), interleukin-1β (IL-1β), interleukin-18 (IL-18), and transforming growth factor-β1 (TGF-β1) (Tschope et al., 2005; Westermann et al., 2007; Rajesh et al., 2012; You et al., 2018). Recent evidence reveals that nucleotide-binding oligomerization domain like receptor (NLR) pyrin domain containing 3 (NLRP3) inflammasome is also a promising molecular marker of diabetes and DCM (Luo et al., 2017).

Curcumin is a potent anti-inflammatory agent and considered beneficial for the amelioration of diabetes and DCM. Curcumin was able to suppress NF-κB signaling pathway, defend against inflammation, cardiac hypertrophy and fibrosis in the heart (He et al., 2015). Curcumin supplementation decreased serum inflammatory factors levels of IL-6, TNF-α and monocyte chemoattractant protein-1 (MCP-1), as well as glucose and glycosylated hemoglobin in diabetic rats. Curcumin incubation also inhibited IL-6, IL-8, TNF-α, and MCP-1 secretion in high glucose-treated monocytes (Jain et al., 2009). Yu et al. (2012) showed that curcumin inhibited AGEs accumulation, decreased inflammatory cytokines of IL-1β and TNF-α, and attenuated diabetes-induced left ventricular dysfunction, cardiomyocyte hypertrophy and interstitial fibrosis in diabetic rats. A similar study reported that curcumin dramatically decreased IL-6 and TNF-α levels in streptozotocin-induced diabetic rats with heart injury (Abo-Salem et al., 2014). A recent study showed that curcumin (300 mg/kg/day for 16 weeks) significantly suppressed collagens deposition in diabetic rat heart, with marked reduction of TGF-β1 production, suppression of type II TGF-β (TβRII) levels and sma- and mad- related protein 2/3 (Smad2/3) phosphorylation, and increment of Smad7 expression in the heart of diabetic rat. They further found that application of curcumin inhibited TGF-β1- or high glucose-induced adenosine monophosphate activated protein kinase (AMPK)/p38 mitogen-activated protein kinase (MAPK) activation in human cardiac fibroblasts *in vitro* (Guo et al., 2018).

**FIGURE 2:**
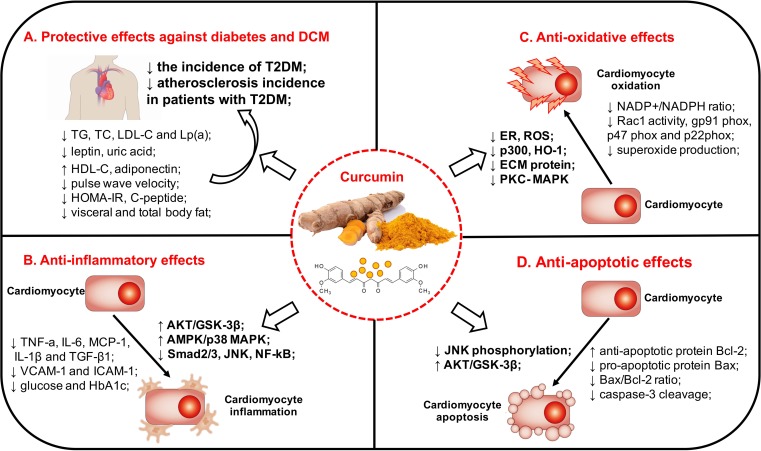
The clinical evidence and potential mechanism by which curcumin might mitigate diabetes and DCM. The pathogenesis of diabetes and DCM is multifactorial, and a plethora of molecular mechanisms have been postulated for the onset and development of diabetes and DCM. Clinical trials have shown that curcumin possessed a potency to decrease blood glucose, improve insulin resistance and ameliorate dyslipidemia in patients with diabetes. Curcumin also exerts a variety of positive effects that was able to attenuate inflammatory activation, oxidative stress, and apoptosis in diabetes and DCM through several signaling pathways. DCM, diabetic cardiomyopathy; T2DM, type 2 diabetes mellitus; DCM, diabetic cardiomyopathy; LDL-C, low density lipoprotein cholesterol; Lp(a), lipoprotein(a); HDL-C, high density lipoprotein cholesterol; HOMA-IR, homeostasis model assessment-insulin resistance; IL-1β, interleukin-1β; IL-6, interleukin-6; IL-18, interleukin-18; TNF-α, tumor necrosis factor-α; TGF-β1, transforming growth factor; ICAM-1, intercellular adhesion molecule-1; VCAM-1, vascular cell adhesion molecule-1; HbA1c, glycosylated hemoglobin A1c; AKT, protein kinase B; GSK-3β, glycogen synthase kinase 3β; AMPK, adenosine monophosphate activated protein kinase; MAPK, mitogen-activated protein kinase; Smad, sma- and mad- related protein; JNK, Jun NH2-terminal kinase; NF-κB, nuclear factor kappa-B; TβRII, type II transforming growth factor-β; MCP-1, monocyte chemoattractant protein-1; NADPH, nicotinamide adenine dinucleotide phosphate; Rac1, Ras-related C3 botulinum toxin substrate 1; HO-1, heme-oxygenase-1; ER, endoplasmic reticulum; ROS, reactive oxygen species; ECM, extracellular matrix; PKC, protein kinase C. **(A)** Curcumin and its protective effects of against diabetes and DCM. **(B)** Curcumin and its anti-inflammatory effects on diabetes and DCM. **(C)** Curcumin and its antioxidant properties on diabetes and DCM. **(D)** Curcumin and its anti-apoptotic effects on diabetes and DCM.

In recent years, curcumin analog has been discovered and widely investigated for their roles in diabetes and DCM. A novel curcumin analog C66 reduces serum and heart hypertriglyceridemia, accompanied by improved cardiac function, inhibition of Jun NH2-terminal kinase (JNK) signaling and cardiac inflammation. They further reported that curcumin analog C66 inhibited a high glucose-induced rise in pro-inflammatory cytokines via inactivation of NF-κB (Wang et al., 2014). J17, another molecule with structural similarities to curcumin, exerts significant inhibitory effects on hyperglycemia-induced inflammation and fibrosis in H9C2 cardiomyocytes and streptozotocin-induced diabetic mouse. The underlying mechanisms may be associated with the inhibition of the P38 and protein kinase B (AKT) signal pathway (Chen et al., 2017). Thus, these studied indicated that curcumin and its analogs can alleviate DCM by attenuating inflammation *in vivo* and *in vitro*. The relevant evidence is summarized in **Table 2**, and the potential mechanism by which curcumin might mitigate diabetes and DCM is exhibited in **Figure 2**.

**Table 2:** Pre-clinical studies about curcumin and its effects on diabetes and DCM.

Animals/cells	Treatments	Main findings	Reference
**Anti-inflammatory effects**			
STZ-induced diabetic SD rats	Curcumin (100 mg/kg/day) for 7 weeks	- Decreased blood levels of TNF-α, IL-6, MCP-1	Jain et al., 2009
		- Decreased glucose and glycosylated hemoglobin	
High glucose-treated monocytes	Curcumin incubation (0.01-1 μM) for 24 h	- Lower TNF-α, IL-6, IL-8, and MCP-1 secretion	Jain et al., 2009
STZ-induced diabetic Wistar rats	Curcumin (100 or 200 mg/kg/day) for 8 weeks	- Attenuated diabetes-induced left ventricular dysfunction, cardiomyocyte hypertrophy and interstitial fibrosis	Yu et al., 2012
		- Inhibited AGEs accumulation	
		- Decreased inflammatory factors (TNF-α and IL-1β)	
		- Activated AKT/GSK-3β signaling pathway	
STZ-induced diabetic Wistar rats	Curcumin (200 mg/kg/day) for 6 weeks	- Inhibited IL-6 and TNF-α levels	Abo-Salem et al., 2014
STZ-induced diabetic SD rats	Curcumin (300 mg/kg/day) for 16 weeks	- Reduced TGF-β1 production	Guo et al., 2018
		- Suppressed TβR II levels and Smad2/3 phosphorylation	
		- Increased Smad7 expression	
High glucose-treated human cardiac fibroblasts	Curcumin incubation (25 μM) for 24 h	- Inhibited TGF-β1- or HG-induced AMPK/p38 MAPK activation	Guo et al., 2018
		- Suppressed collagen synthesis in the fibroblasts	
STZ-induced diabetic C57BL/6 mice	Curcumin (5 mg/kg/day) for 3 months	- Reduced hypertriglyceridemia in both serum and hearts	Wang et al., 2014
		- Improved cardiac function, inhibition of JNK signaling and cardiac inflammation	
		- Inhibited a high glucose-induced rise in pro-inflammatory cytokines via inactivation of NF-κB	
STZ-induced diabetic C57BL/6 mice	Curcumin analog, J17 (10 mg/kg/day) for 42 days	- Suppressed hyperglycemia-induced inflammation, hypertrophy and fibrosis	Chen et al., 2017
		- Decreased TNF-α and ICAM-1	
High glucose-treated H9C2 cardiomyocytes	Curcumin analog, J17 (2.5 or 10 μM) for 30 min	- Decreased pro-inflammatory cytokines (TNF-α and IL-6) and adhesion molecules (VCAM-1 and ICAM-1) expressions	Chen et al., 2017
		- Decreased AKT phosphorylation	
		- Inhibited the HG-induced increase in fibrotic genes (collagen-IV, TGF-β, and collagen-I)	
**Antioxidant properties**			
STZ-induced diabetic Wistar rats	Curcumin (100 or 200 mg/kg/day) for 8 weeks	- Attenuated NADP+/NADPH ratio, Rac1 activity and the expression of NADPH oxidase subunits of gp91 phox, p47 phox	Yu et al., 2012
STZ-induced diabetic Wistar rats	Curcumin (200 mg/kg/day) for 6 weeks	- Restored cardiac antioxidant enzymes (catalase, superoxide dismutase, and glutathione-*S*-transferase)	Abo-Salem et al., 2014
STZ-induced diabetic SD rats	Curcumin (100 mg/kg/day) for 8 weeks	- Decreased NADPH oxidase subunits (p67phox, p22phox, gp91phox)	Soetikno et al., 2012
		- Decreased the mRNA expression of transcriptional co-activator p300 and atrial natriuretic peptide	
		- Decreased accumulation of ECM protein	
		- Reversed the increment of superoxide production	
STZ-induced diabetic C57BL/6 mice	Curcumin (5 mg/kg/day) for 3 months	- Protection against diabetes-induced cardiac fibrosis, oxidative stress, and ER	Wang et al., 2014
STZ-induced diabetic rats	Curcumin (20 mg/kg/day) for 45 days	- Prevented diabetes-induced upregulation of HO-1 expression and activity	Aziz et al., 2013
**Anti-apoptotic effects**			
STZ-induced diabetic Wistar rats	Curcumin (100 or 200 mg/kg/day) for 8 weeks	- Prevented diabetes-induced cardiomyocytes apoptosis	Yu et al., 2012
STZ-induced diabetic C57BL/6 mice	Curcumin (5 mg/kg/day) for 8 weeks	- Prevented high glucose-induced apoptosis in cardiomyocytes and the development of diabetic cardiomyopathy	Pan et al., 2014
		- Inhibition of JNK phosphorylation	
STZ-induced diabetic C57BL/6 mice	Curcumin (5 mg/kg/day) for 3 months	- Protection against diabetes-induced cardiac fibrosis, oxidative stress, and ER;	Wang et al., 2014
High glucose-treated neonatal rat cardiomyocytes	Curcumin incubation (10 μM) for 24 h	- Inhibited the increased Bax/Bcl-2 ratio elicited by high glucose exposure	Yu et al., 2016
		- Increased AKT and GSK-3β phosphorylation	

*DCM, diabetic cardiomyopathy; STZ, streptozotocin; SD, Sprague-Dawley; HG, high glucose; AGEs, advanced glycation end products; ICAM-1, intercellular adhesion molecule-1; VCAM-1, vascular cell adhesion molecule-1; IL-1β, interleukin-1β; IL-6, interleukin-6; IL-18, interleukin-18; TNF-α, tumor necrosis factor-α; TGF-β1, transforming growth factor; NF-κB, nuclear factor kappa-B; TβRII, type II transforming growth factor-β; Smad, sma- and mad-related protein; MCP-1, monocyte chemoattractant protein-1; AMPK, adenosine monophosphate activated protein kinase; MAPK, mitogen-activated protein kinase; JNK, Jun NH2-terminal kinase; AKT, protein kinase B; NADPH, nicotinamide adenine dinucleotide phosphate; Rac1, Ras-related C3 botulinum toxin substrate 1; PKC, protein kinase C; HO-1, heme-oxygenase-1; GSK-3β, glycogen synthase kinase 3β; ECM, extracellular matrix; ER, endoplasmic reticulum.*

### Curcumin and Its Antioxidant Properties on Diabetes and DCM

Oxidative stress is known to participate in the development of diabetes and DCM. Reactive oxygen species (ROS) have been postulated to play a significant role in the pathogenesis of diabetes and DCM. Anderson et al. firstly showed the evidence of increased oxidative stress, with higher mitochondrial hydrogen peroxide (H_2_O_2_) production and elevated 4-hydroxynonenal- and 3-nitrotyrosine-modified proteins levels in atrial tissue in human diabetic hearts (Anderson et al., 2009). Furthermore, Ye et al. (2004) provided the evidence of causality between ROS and DCM. It indicated that overexpression of catalase or manganese SOD partially restored the dysfunction of mitochondrion and cardiomyocyte contractility in diabetic mice.

Curcumin was able to act as a natural free radical scavenger as its chemical structure and has anti-oxidative potency. Abo-Salem et al. (2014) found that curcumin mitigated the reduction of cardiac antioxidant enzymes and glutathione levels in diabetic rats, including SOD, catalase, and glutathione-*S*-transferase. It showed that curcumin attenuated nicotinamide adenine dinucleotide phosphate (NADPH) oxidase subunits (p47phox and gp91phox) expressions, NADP+/NADPH ratio, and Ras-related C3 botulinum toxin substrate 1 (Rac1) activity in diabetic rats (Yu et al., 2012). Soetikno et al. (2012) also showed that curcumin treatment markedly decreased NADPH oxidase subunits (p67phox, p22phox, gp91phox) expressions and superoxide production in diabetic rats, possibly by inhibiting protein kinase C (PKC)- MAPK signaling pathway. The novel curcumin analog C66 was able to inhibit JNK activation in diabetes, resulting in protection of endoplasmic reticulum (ER) and oxidative stress against induced by diabetes (Wang et al., 2014). Aziz et al. (2013) reported that curcumin protected against diabetes-induced heme-oxygenase-1 (HO-1) upregulation in the cardiac tissue of diabetic rats.

In addition, the heart depends on continuous mitochondrial ATP supply and maintained redox balance to properly develop force. However, myocardial energetic-redox balance is perturbed exposed to hyperglycemia, that is a critical driver of mitochondrial dysfunction in the diabetic myocardium (Aon et al., 2015). Mitochondria control cell respiration and energy production are also closely related to oxidative stress. Wright et al. (2011) found reduced cardiac function and increased myocardial oxygen consumption in *db/db* diabetic mice, that was possibly regulated by increased mitochondrial ROS generation and lipid and protein peroxidation products in mice hearts. Alleviating oxidative stress targeting mitochondrial function has been confirmed as a potential therapeutic approach to limit ischemia-reperfusion (IR)-induced cardiac injury and protect against diabetes (Yang et al., 2013). Thus, preserving mitochondrial function, especially maintaining mitochondrial redox potential is also an important mechanism underlying the protective effects of curcumin against oxidative stress and diabetes. It showed that curcumin significantly decreased palmitate-induced oxidative stress and increased mitochondrial permeability, thus it was able to promote glucose-induced insulin secretion in pancreatic β-cells (Hao et al., 2015a). Lin et al. (2014) showed that collagen fibers in interstitium and expansion of mitochondria in cytoplasm of myocardium were increased in diabetic rats, that can be ameliorated by curcumin derivative B06 treatment. However, studies about direct effects targeting mitochondrial function of curcumin against DCM are limited, and further investigations about curcumin and its role in preserving mitochondrial function in DCM are needed. Thus, these studied indicated that curcumin can play its antioxidant properties in diabetes and DCM (**Table 2** and **Figure 2**).

### Curcumin and Its Anti-apoptotic Effects on Diabetes and DCM

Higher degree of cell apoptosis can be observed in the hearts of diabetic patients and in rodent models of diabetes. It has been shown that renin–angiotensin–aldosterone system (RAAS) activation, increased production of ROS, and abnormal expressions of apoptosis-related molecules are all associated with cardiomyocyte apoptosis and necrosis in diabetic hearts (Frustaci et al., 2000). Yu et al. (2012) found that curcumin supplementation (200 mg/kg/day) for 8 weeks prevented DM-induced cardiomyocytes apoptosis, evaluated by increased TUNEL-positive cells in diabetic rats. Pan et al. (2014) demonstrated that curcumin analog mitigated high glucose-induced apoptosis in cardiomyocytes and prevented the development of DCM, through the inhibition of JNK phosphorylation in the diabetic heart (Ren and Sowers, 2014). Wang et al. (2014) indicated a marked decrease in anti-apoptotic protein (Bcl-2), as well as an increase in caspase-3 cleavage and pro-apoptotic protein (Bax) in diabetic mice. However, treatment with curcumin analog (5 mg/kg/day) for 3 months significantly reversed the diabetes-induced cardiac cells apoptosis in diabetic mice (Wang et al., 2014). It also indicated that curcumin supplement effectively inhibited the elevated Bax/Bcl-2 ratio and alleviated high glucose-induced cardiomyocyte apoptosis in neonatal rat cardiomyocytes, accompanied by increased AKT and glycogen synthase kinase 3β (GSK-3β) phosphorylation (Yu et al., 2016). Thus, the evidence indicated that curcumin can alleviate cardiomyocyte apoptosis in diabetes (**Table 2** and **Figure 2**).

## Conclusion

In summary, as a natural compound, curcumin plays a critical role in human health. Clinical trials and preclinical studies have shown that curcumin possessed a potency to decrease blood glucose, improve insulin resistance and ameliorate dyslipidemia in patients with diabetes. It can exert positive effects on attenuate inflammatory activation, oxidative stress, and apoptosis in diabetes, and it is proposed to have the potential impacts to protect against DCM. Curcumin, which is believed to be pharmacologically safe, effective, and with low adverse effects, it can be considered as a promising agent for alternative therapies for the prevention and treatment of diabetes and its cardiovascular complications. However, clinical trials about the long-term effects and precise mechanisms of curcumin on diabetes and DCM in humans are still lacking. Thus, more randomized controlled trials and pre-clinical experiments are required to confirm the efficacy of curcumin and to elucidate the various mechanisms by which curcumin might mitigate diabetes and DCM, which may have the potential to open new horizons in the early prevention and treatment of the development of diabetes and DCM in the future.

## Author Contributions

JZ and JC collected and synthesized the data and wrote the manuscript. QF and SZ reviewed and edited the manuscript. JZ and XX contributed to the design of this review.

## Conflict of Interest Statement

The authors declare that the research was conducted in the absence of any commercial or financial relationships that could be construed as a potential conflict of interest.
